# Anæsthetics in Dental Practice

**Published:** 1888-12

**Authors:** H. C. Raymond

**Affiliations:** Milan, Mich.


### Anæsthetics in Dental Practice.

BY H. C. RAYMOND, D.D.S., MILAN, MICH.

Having read the papers published in the DENTAL REGISTER of September and October, anent the above; I beg to offer a few remarks on the use of nitrous oxide gas in dental practice, for I believe it to be *par exeellence* the anæsthetic for dental use in the majority of cases.

What are the objections urged against it by those who would have us resort to the use of more dangerous agents?

1. Its transient effect. This I admit forms ground for an objection, but is it so serious as many of the votaries of other agents would have us believe? I think not! In this period of dental advancement the necessity for wholesale extraction is fast dying away, so that lengthy operations in that direction are the exception, not the rule.

I certainly think that nitrous oxide is not too transient an agent for the greater number of cases coming under our care. I know that occasionally the removal of a single tooth will take a longer time than the nitrous oxide allows; but such cases are rare indeed, and when they do appear it will be soon enough to resort to the use of other agents.

If it is necessary to remove a great number of teeth, it is not an indication that chloroform or ether are the only or the best agents to be used. Nitrous oxide, unlike other agents may in most cases, be administered more than once on the same day, or on consecutive days, and that too, without being attended by the unpleasant results which oftener than not follow a single administration of ether or chloroform, so that in nearly every case even the longest of operations can be performed most successfully under the influence of nitrous oxide. There are few, if any intelligent persons who would not rather take the nitrous oxide several times, than take ether or chloroform once, knowing the danger and unpleasant after effects attending the use of them.

2. Objection put forward against its use is failure to produce anæsthesia, such failure being stated to result, 1st, from excitement on the part of the patient, who will not have the gas administered. 2d, owing to the idiosyncrasies of the patient. The first stated cause has not a leg to stand upon. I should say that the cause is the inability on the part of the operator, either from not understanding how to administer the agent, or from want of necessary assistance during the administration; both faults would cause just as unsatisfactory a result with the use of other agents as with nitrous oxide.

The second, idiosyncrasies, is a term I am inclined to believe behind which many seek to hide their failures. Dr. Garretson quotes Dr. J. B. Flagg as follows: He (Dr. Flagg) "believed in no idiosyncrasies except it might be a very high nervous temperament, and that seeming idiosyncrasies might generally be explained by an examination of the operator's ignorance of the agent." Possibly such conditions do exist, but they are extremely rare, and are no more likely to be encountered with the use of nitrous oxide than with other agents. Some of our operators of largest experiénce tell us that they have never met such cases. We know that some are more susceptible to it than others, but no ordinary constitution can successfully resist the influence of *pure gas properly administered.*

There is no doubt that nitrous oxide is the safest of all anæsthetics now in use, it has without doubt been used the greatest number of times, and by far the fewest deaths have resulted from its use, indeed, it is doubtful if it has caused any deaths, when used in the pure state, for " the few deaths that have followed its use occurred during the time that the gas was made in the office of the one using it."* Apart from its great safety it has many recommendations as an anæsthetic, it is pleasant to take, with no sense of suffocation, it produces anæsthesia rapidly, with little or no excitement, and the patient, after a quick recovery from its effects, feels, in nearly all cases, well and strong, unaccompanied by that feeling of nausea, which nearly always attends the use of other agents.

Its use is contra-indicated only in very few cases, and it may be used with safety where the stronger agents would be extremely dangerous.

Though it is not my aim to pose as instructor in its administration, I think I might mention a detail connected with it which is worthy of consideration. I think it very unwise to begin to talk to a patient when only partly recovered from its influence, he should not be bothered with questions, and friends should not be allowed to ask, immediately after the operation, (as they will do, if not cautioned or kept out of the way), if he felt it, for as a rule the patient is just in that state when he is apt to think the worst of everything, and, if during anæsthesia he was only insensible to pain, and not unconscious (a state common when under the influence of nitrous oxide), he will, nearly in every case, say he felt it. Hence the declaration of fraud mentioned in Dr. Harris' paper—whereas, were he not spoken to till the effects of the gas had passed off, he would give quite a different verdict.

I do not say that dentists should never use stronger anæsthetics than nitrous oxide, but I think they are used very often unnecessarily, and where the safer agent would do equally as well. I think it would be better for our profession if ether and chloroform were only used, when absolutely necessary, and then they should be given by a medical man experienced by their use.

Nitrous oxide may be administered by the dentist himself, without the aid of an M. D. It is our anæsthetic—it is the adopted one of the dental profession, and is most desirable for all minor operations in the mouth. In the hands of the careful administrator, and skillful operator, it has proved to be one of the most reliable, and the safest of all anæsthetics at present known.

